# Educational intervention in social skills for Primary Care nurses

**DOI:** 10.1590/0034-7167-2022-0503

**Published:** 2023-10-09

**Authors:** Aline Loiola Moura Bianconi, Tatiana da Silva Melo Malaquias, Adriana Valongo Zani, Elen Ferraz Teston, Maira Sayuri Sakai Bortoletto, Maria do Carmo Fernandez Lourenço Haddad

**Affiliations:** I Universidade Estadual de Londrina. Londrina, Paraná, Brazil; II Universidade Estadual do Centro-Oeste. Guarapuava, Paraná, Brazil; III Universidade Federal do Mato Grosso do Sul. Campo Grande, Mato Grosso do Sul, Brazil

**Keywords:** Nursing, Health Management, Interpersonal Relations, Primary Health Care, Social Skills, Enfermaría, Gestión en Salud, Relaciones Interpersonales, Atención Primaria de Salud, Habilidades Sociales, Enfermagem, Gestão em Saúde, Relações Interpessoais, Atenção Primária à, Saúde, Habilidades Sociais.

## Abstract

**Objective:**

to assess an educational intervention on social skills for nurses who work in Primary Health Care.

**Method:**

a qualitative research-intervention study, carried out in the municipalities covered by the 17^th^ Health Regional of Paraná. It was developed in three interrelated stages: exploratory, where meetings were held with the managers to define the groups and logistics for running the course; intermediate, developed in meetings with different groups of nurses, addressing selected topics; assessment, in which the nurses developed a personal plan to improve their social skills.

**Results:**

participants were 57 nurses who acted as coordinators of Primary Care. They considered the educational intervention in social skills fundamental for positive changes in their professional performance.

**Final considerations:**

the educational intervention in social skills was assessed as an important strategy to strengthen the development of nurses’ managerial and care skills.

## RESUMO

**Objetivo:**

avaliar uma intervenção educativa em habilidades sociais para enfermeiros que atuam na Atenção Básica à Saúde.

**Método:**

trata-se de uma investigação qualitativa do tipo pesquisa-intervenção, realizada nos municípios de abrangência da 17ª Regional de Saúde do Paraná. Foi desenvolvida em três etapas inter-relacionadas: exploratória, onde foram realizadas reuniões com os gestores para definição dos grupos e logísticas de funcionamento do curso; intermediária, desenvolvida nos encontros com os grupos distintos de enfermeiros, abordando os temas selecionados; avaliativa, na qual os enfermeiros elaboraram um plano pessoal para aprimorarem suas habilidades sociais.

**Resultados:**

os participantes foram 57 enfermeiros que atuavam como coordenadores da Atenção Básica. Consideraram a intervenção educativa em habilidades sociais fundamental para mudanças positivas na sua atuação profissional.

**Considerações finais:**

a intervenção educativa em habilidades sociais foi avaliada como uma estratégia importante para fortalecer o desenvolvimento das competências gerencias e assistenciais do enfermeiro.

## RESUMEN

**Objetivo:**

evaluar una intervención educativa sobre habilidades sociales para enfermeros que actúan en la Atención Primaria de Salud.

**Método:**

se trata de una investigación cualitativa del tipo investigación-intervención, realizada en los municipios de actuación de la 17ª Regional de Salud de Paraná. Se desarrolló en tres etapas interrelacionadas: exploratoria, donde se realizaron reuniones con los directivos para definir los grupos y la logística para la operación del curso; intermedio, desarrollado en encuentros con diferentes grupos de enfermeros, abordando temas seleccionados; evaluativo, en el que las enfermeras desarrollaron un plan personal para mejorar sus habilidades sociales.

**Resultados:**

participaron 57 enfermeros que actuaban como coordinadores de Atención Primaria. Consideraron la intervención educativa en habilidades sociales fundamental para cambios positivos en su desempeño profesional.

**Consideraciones finales:**

la intervención educativa en habilidades sociales fue evaluada como una estrategia importante para fortalecer el desarrollo de habilidades gerenciales y asistenciales de los enfermeros.

## INTRODUCTION

Primary Care (PC) can be defined as a set of health actions at the individual and collective level that encompasses health promotion and protection, prevention, diagnosis, treatment of injuries and rehabilitation. It is conceived as a care coordinator and organizer of the regionalized Health Care Network (RAS - *Rede de Atenção à Saúde*)^(1)^.

To qualify and humanize the service, it is essential to identify the general difficulties that may be present in the work process, such as insufficient material resources and equipment, reduced number of professionals, political influence in management, among others^(2)^.

In addition to these problems, interpersonal relationships between managers and the health team stand out, which cause many conflicts, sometimes difficult to negotiate and resolve. In this regard, professionals who act as leaders in this work process need to offer quality care to the local population, in addition to accountability in meeting targets for the Ministry of Health^(2)^.

Therefore, interdisciplinary health teams face challenges in their professional practice, as well as in the service management process for constructing a transforming action, with the aim of developing expanded health visions that allow creating proposals and transformations of multidisciplinary practices^(3)^.

In this context, nurses contribute significantly with their knowledge, as their work process is directed towards the various dimensions of clinical practice, such as direct and indirect care, teaching, research, management, health promotion and disease prevention, treatment and rehabilitation. From this perspective, nurses represent the main actor in health actions, as they have assumed leadership positions in current scenarios that can define the quality of care provided^(2-3)^.

Nurses are the nursing team managers, in addition to articulating the activities of several other professionals in the health team, having to develop their potential to expand the skills relevant to the work process management, especially in communication, interpersonal relationships and in the development of a climate conducive to the exercise of leadership^(4)^.

In clinical practice, it is observed that the training of this professional must be permanently complemented and, in order to achieve the objectives of this modality of health care, constant approximation between people in the context of social interactions is necessary. However, some elements of these relationships need to be understood and developed so that interactions are friendly and resolving, highlighting social skills^(4-5)^.

Social skills (SS) refer to social behaviors valued in a given culture, with a high probability of favorable results for individuals, their group and the community, and which can contribute to a socially competent performance in interpersonal tasks^(5)^.

The main SS classes highlighted by Del Prette and Del Prette^(6)^ are: communication skills (asking and answering questions; initiating, maintaining and ending a conversation; praising); civility skills (saying thanks; asking please; introducing oneself); assertive coping skills (expressing an opinion; making and refusing requests; dealing with criticism; dealing with different opinions; interacting with authorities); empathic and positive feeling expression skills (being friendly; expressing joy); social work skills (speaking in groups; coordinating groups; dealing with leaders); educational SS (from parents; teachers).

Caballo^(7)^ states that such skills can be innate or acquired through training and practice in interpersonal relationships. Thus, it is possible that a person with low competency in SS can competently present and perform a given social task.

Interventions in SS are strategies that aim to prevent future behavioral and relational difficulties. Such intervention programs aim to teach new significant skills and decrease or extinguish competing behaviors^(6)^.

In the daily practice of nurses who work in PC, due to the work dynamics and the diversity of actions, there is a constant need to deepen and discuss SS.

## OBJECTIVE

To assess an educational intervention on SS for nurses who work with PC.

## METHODS

### Ethical aspects

The study followed the precepts of Resolution 466/12 of the Brazilian National Health Council and was submitted and approved by the Research Ethics Committee.

### Study design and place

This is a qualitative research-intervention study, which is carried out together with the researched population, aiming at the procedural modification of the object under study, making use of influences in everyday life^(8)^. It was conducted and structured with reference to the COnsolidated criteria for REporting Qualitative research (COREQ) checklist^(9)^.

The study was carried out in the host municipality of the 17^th^ Health Regional of Paraná, with managers and coordinating nurses of the PC units who worked in the 21 municipalities covered by this regional.

The state of Paraná is subdivided into four health macro-regions: East Macro-regional, West Macro-regional, North Macro-regional and Northeast Macro-regional, which in turn are subdivided into 22 regional. The 17^th^ Health Regional is part of the North Macroregional, has Londrina as its seat municipality and is responsible for supporting the 21 municipalities in the health area as well as in the management of regional issues, promoting the continuous search for efficiency in quality care provision.

### Study participants

Study participants consisted of five managers from the 17th Regional Health of Paraná and 75 nurses who acted as PC coordinators in municipalities belonging to this regional. The selection of nurses was intentionally carried out by the managers, but only 57 participated in the educational intervention. The 18 absentees justified their absences, of which 10 had difficulties with transportation, two were on vacation and six were unable to participate due to personal problems.

### Data collection and organization

**Figure 1:**
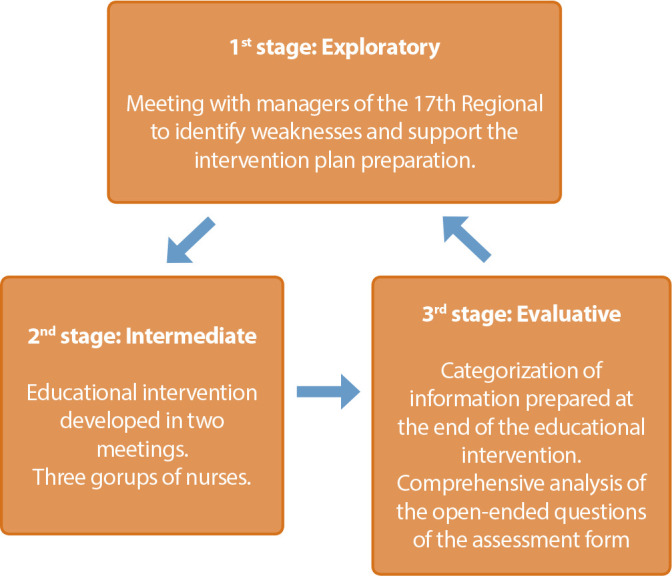
Operational stages of the intervention-research, Londrina, Paraná, Brazil, 2021

The intervention research was developed in three interrelated stages, namely: exploratory, intermediate and evaluative, as shown in Figure 1.

In the exploratory stage, three meetings were held with the 17^th^ Health Region managers, who requested training on this topic for nurses who held the position of coordinators in the municipalities that make up this health region. In these meetings, the constitution of the three groups, the objectives and the course load were defined as well as the topics selected to be addressed. Managers chose not to participate in the course to ensure freedom of expression for nurses.

In the intermediate stage, developed with the 57 nurses, topics were addressed through active pedagogical methodologies, including expository dialogue classes and group dynamics, as described below:

**SS:** concept and differentiation of SS and social competency.

**Classes of SS:** SS and applicability in the managerial practice of nurses with a focus on interpersonal relationships.

**Self-monitoring social skill:** self-knowledge to develop self-monitoring; self-monitoring as a facilitator of nurse leaders’ assertiveness.

**Social communication skills and feedback:** communication as an essential social skill for care management; feedback as a management tool.

**Social work skills and interpersonal relationships:** problem-solving; decision-making; conflict mediation; negotiation techniques.

**Difference between generations:** leadership styles: decision-making; nurses’ leadership in the scenario of nursing and the health area.

**Process of change:** experience about change; how to adapt the leadership style to current demands.

Participants were divided into three distinct groups, with approximately 20 nurses in each, who received the same content covered. Two meetings were held with each group, with a workload of 16 hours of training per group.

The first meeting addressed the concepts of SS, performance and social competency, and the interfaces between SS and nursing care management. Examples of the differences between empathic and pro-empathic communication were presented as well as reflections and discussions about empathic communication for leadership. In these workshops, the nurses had the opportunity to dramatize the SS of giving and receiving feedback and their intersection with other SS.

In the second meeting, current leadership styles were addressed, reflections were made on the SS required for leadership, explaining the specificities of social work skills in professional practice and discussing the SS involved in the following topics: planned change, conflict mediation, negotiation and decision-making.

In the evaluative stage, nurses were given the opportunity to develop a personal plan to improve their SS, containing three dimensions: 1) What social skills will I fail doing; 2) What social skills will I continue to practice; 3) What social skills will I start. The personal plan was developed individually and then discussed in groups, which synthesized and grouped the information for presentation in the large group, as indicated in the results stage.

The educational intervention assessment took place individually, through the application of an assessment form prepared by the authors, consisting of four dimensions, referring to the content addressed, the instructor performance, the relationship between participants and general training assessment. Each dimension had subsections and categorization for responses such as excellent, good, reasonable, insufficient and not applicable. At the end of the form, nurses also answered the question: how will this intervention contribute to your professional performance? Comment your answer.

### Data analysis

The contents described in the personal plans of the three groups were grouped and analyzed according to Bardin’s technique, following the three phases proposed by the author: 1. pre-analysis; 2. material exploration; and 3. treatment of results: inference and interpretation^(10)^.

The presentation of the categories and subcategories identified in participants’ responses has examples of excerpts from the statements coded as A1 to A21, B1 to B12, and C1 to C24 (nurses from groups A, B and C), in order to maintain anonymity.

The data from the assessment form were presented using a table, with absolute and relative frequency, and participants’ opinions about the educational intervention were grouped by similarity.

## RESULTS

A total of 57 nurses who worked as PC coordinators in the 21 municipalities that comprise the 17^th^ Health Regional of Paraná participated in the meetings. Among these professionals, 96.4% were women, 31.6% aged between 21 and 30 years old, 36.8% between 31 and 40 years old, 22.8% between 41 and 50 years old, and 8.8% were over 51 years old. Of the nurses who participated in the intervention, 78.9% had specialization and, among these, 33.3% in the area of public health.

The categorization of each participant’s personal plan for improving their SK allowed listing four categories of desire for change and progress, as shown in Chart 1.

**Chart 1:** Summary of the four categories listed in participants’ personal plan to improve their social skills, Londrina, Paraná, Brazil, 2021

Categories	Subcategory 1: Keep doing	Subcategory 2: Fail to doing	Subcategory 3: Start doing
Category 1: Desire for progress in self-knowledge and self-monitoring	Dealing with criticism; Reflecting feelings; Admitting failures; Self-monitoring emotions; Reflecting my professional practice.	Not dealing with criticism; Caring about what others think; Criticizing based on my opinion; Blaming myself; Acting mechanically; Prejudging; Acting with anxiety.	Admitting failures; Dealing with criticism; Seeing in me a leader; Observing others’ work to improve mine; Stopping caring about other people’s comments.
Category 2: Desire for progress in communication	Expressing support; Establishing a good dialogue; Asking for feedback; Making friends and saying thanks; Praising; Making team meetings with space for discussions; Expressing opinions, civility (saying good morning, saying hello); Being thankful; Proposing innovations.	Complaining; Blaming others; Giving satisfaction; Interrupting people during their speeches; Talking more and listen less; Aggressive communicating; Doing what belongs to the other; Being afraid to lead; Mixing friendship with work; Expressing anger.	Giving and asking for feedback; Making eye contact during conversations; Listening more to the team and praise; Saying good morning; Policing for non-verbal language; Doing active listening; Accepting or declining orders; Interacting with authority; Expressing displeasure; Expressing my opinion; Fixing individually; Recognizing team work; Learning to say no; Exchanging experience for decision-making.
Category 3: Desire for progress in empathy and expression of positive feelings	Cultivating love; Practicing empathy.	Underestimating the other’s capacity; Letting friendship interfere with the work process; Being more reason and less emotion in decision-making; Being emotional.	Expressing support; Impersonal service; Avoiding conflict; Cultivating love.
Category 4: Desire for advancement in leadership	Speaking in public; Decision-making; Planning the work process; Mediating conflicts; Exercising democratic leadership; Using appropriate tools to achieve goals; Establishing criteria for decision-making; Reassessing the work process; Recognizing teamwork and the importance of a leader; Updating myself.	Failure to meet goals; Procrastinating; Expressing to people that “in my time it was like this”; Seeing things as difficulty.	Asking for behavior change; Coordinating groups, Public speaking, organizing; Imposing myself more with people; Delegating more; Conducting periodic meetings and training with the team; Ensuring that the team meets schedules; Developing an instrument for assessing the work process.

Participants considered the educational intervention as excellent and good in the general assessment, in the four dimensions, as shown in Table 1.

**Table 1:** General assessment of the educational intervention in social skills by participants, Londrina, Paraná, Brazil, 2021

Dimensions assessed	Great (n / %)			Good (n / %)			Reasonable (n / %)	Insufficient (n / %)	Not applicable (n / %)
Topic addressed	57	100.0		-			-	-	-
Instructor performance	55	96.4		02	3.6		-	-	-
Relationship between participants	45	78.9		12	21.1		-	-	-
General training assessment	48	84.2		09	15.8		-	-	-

Caption: n = absolute number of answers.

Below are some of participants’ answers reported in the open-ended question of the assessment form:

*It makes us self-aware, teaches us to think before acting, remember the role of leader and improve our performance.* (B1)

*A negative point in the intervention was messing with our feelings and our weaknesses.* (C3)

*This intervention will contribute to my professional life, because by changing my being, I will relate and communicate better with my team.* (A6)

*Through this intervention, I am already changing the way I listen to people before judging them.* (B3)

*The social skills addressed practically improved my performance as a team leader.* (C12)

The five managers of the 17^th^ Health Region, who supported the elaboration of a plan for this intervention, participated in the course closing, which was considered an excellent opportunity to understand the educational process results and hear participants’ opinion regarding their future plans as well as a way to demonstrate nurses’ commitment to improving SS in their work process.

Thus, the desire to continue the educational meetings prevailed in nurses’ speeches, and to continue the process as continuing education was a participants’ proposal expressed to their managers, according to the reports below:

*This course should be monthly and be expanded to all coordinators and nurses in our regional office.* (B8)

*The course made me realize that working on social skills through dynamics favors group understanding and enables applicability in our workplace. I hope this course is offered to other nursing professionals in the Health Care Network.* (C23)

*I would like to have other modules of the course so that we can continue the process of improving social skills.* (A18)

## DISCUSSION

The educational intervention on SS was perceived by PC nurses as something positive in the development of their professional practice. This perception is also identified in a study with nurses from the Family Health Strategy in southern Brazil^(11)^, who considered the educational intervention as a fundamental strategy of permanent education for the improvement of their managerial skills.

The need for improvement in SS demonstrates how essential the constant search for updates and training related to their communication and relational skills is. Appropriately trained nurses are better able to teach their team to provide qualified care that meets users’ and families’ health needs^(12)^.

Continuing education, through educational interventions, must be facilitated and inserted in nurses’ daily lives so that their managerial and care skills are strengthened, reflecting on a qualified practice with assertive decisions^(13)^.

In nurses’ professional practice, sensitivity is needed to accommodate the demand and subjectivity of each team member. Therefore, it is constantly recommended to search for feeling perception and appreciation, thoughts and history of the other, taking care to know him as a whole and to know each other. The team leader should seek to develop self-knowledge as well as relational skills, focusing on assertive communication and teamwork^(14)^, as expressed by participants during the course.

A US study conducted during the first wave of the COVID-19 pandemic with nurses caring for hospitalized SARS-CoV-2 infected patients, demonstrated that the effective communication of nurses and their team was fundamental to transmit critical information and positively impacted the care of patients and their families, especially in that moment of crisis and global health emergency that they were experiencing^(15)^.

The leadership process requires responsibility, common sense, self-knowledge and self-monitoring, which must be improved throughout the career in order to exercise the profession with coherence and security.

Self-monitoring is a complex skill and comprises different behaviors, such as identifying response alternatives and predicting their possible consequences, recognizing social encouragement and responding differentially to them, making decisions, self-control behavioral expressions (both emotional and cognitive) and finding more effective coping strategies to better manage daily life^(16)^.

For Del Prette and Del Prette^(6)^, self-monitoring promotion is essential for the performance of SS as well as for the social competency practice, especially for team leaders. It is an investment that can have a positive impact on individuals’ development, so it should be promoted from childhood, considering behavioral indicators that are possible to be observed at this stage of life.

However, for self-knowledge it is necessary to develop the ability of self-monitoring. Such skills are essential for individuals to manage their self-presentation, model their actions according to the situation they are experiencing, regardless of the sector in which they are inserted^(6)^.

It was observed, in this study, that a significant portion of participants pointed out that identifying their weaknesses was a negative point in the educational process; however, recognizing their weaknesses and strengths is a basis for decision-making, since it is through self-knowledge that professionals understand what makes sense to them.

In this regard, it can coherently define what is most appropriate according to their values and objectives. Thus, it was considered as a positive aspect of the educational intervention the encouragement to self-recognition of weaknesses, because only by identifying these aspects will the person be able to develop strategies to improve their relationships and communication.

Communication and leadership skills are intertwined, they should be encouraged during academic life and continued throughout the career, as this contributes to constructing more reflective, creative, critical professionals, capable of expressing opinions, making decisions in conflicting situations of everyday life, in addition to enhancing the care provided to internal and external clients of health services^(17)^.

The identification by participants of the need for improvement in empathy and the expression of positive feelings demonstrates that interacting socially has become one of the essential skills, currently, in organizations. In order to achieve good results, and to understand leaders’ real role, it is necessary to recognize their SS and competencies, and understand the difference between the one who leads and the one who just manages^(18)^.

It was evident, in participants’ speeches, the relevance of encouragement regarding teaching and reflection on the leadership process in nurses’ practice already inserted in their work. Like other managerial skills, SS are extremely relevant to nurses’ interpersonal competency, and must be constantly developed through permanent education. Due to the fact that nurses exercise a leadership position, it is essential that these professionals are competent in relational processes and, for this, they must have a SS repertoire^(19)^.

Interpersonal competency improvement, through educational interventions, can facilitate work relationships and provide problem-solving in professional practice. Therefore, it is necessary to build a SS repertoire for nurses in their relationship with work teams, considering that they have the ability to influence their behavior, with a view to fulfilling the objectives of the service and, consequently, achieving patients’ well-being^(1)^.

### Study limitations

A third meeting was initially planned to assess the implementation of a personal development plan. However, it could not be carried out due to the dengue outbreak in the region as well as due to the onset of the COVID-19 pandemic in March 2020, making it impossible to identify whether the strategies mentioned by nurses were inserted in their professional practice.

### Contributions to nursing

The results of this study demonstrated that educational interventions should be strengthened in nurses’ practice and that the development of SS in professional interactions, as well as in nurses’ daily interactions, need to be in constant improvement and need facilitators to achieve this objective through new scientific research, implementation of new personal training practices and a new organization of services and work processes.

For future investigations, it is important to extend the approach to all nursing team professionals involved in health care and who are also part of the management process, like other nurses, technicians and nursing assistants, broadening the view on educational interventions linked to SS.

## FINAL CONSIDERATIONS

The educational intervention in SS for coordinating nurses of PC in the health region under study was considered a fundamental strategy for developing managerial skills and improving professional practice, highlighting the need to implement a management model that benefits the development of skills in the field of human relations.

It is hoped that the results of this study will enable the development of better interpersonal practices and the achievement of service objectives and may positively influence the relationships on which professionals’ actions are built.
